# Supplementary material for: RNF149 Promotes HCC Progression through Its E3 Ubiquitin Ligase Activity
Source: Cancers (Basel). 2023 Oct 29;15(21):5203. doi: 10.3390/cancers15215203 (PMC10648572; doi:10.3390/cancers15215203)

Supplementary Materials

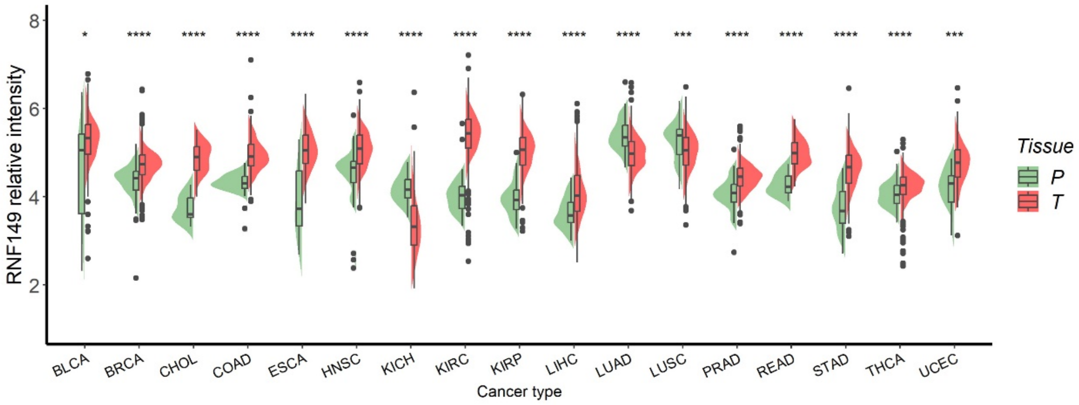

Supplementary Figure S1. The expression of *RNF149* in pan-cancer. TCGA transcriptomics data were used to analyze the expression pattern of RNF149. $^{*}P < 0.05$, $^{***}P < 0.001$,$^{****}P < 0.001$.

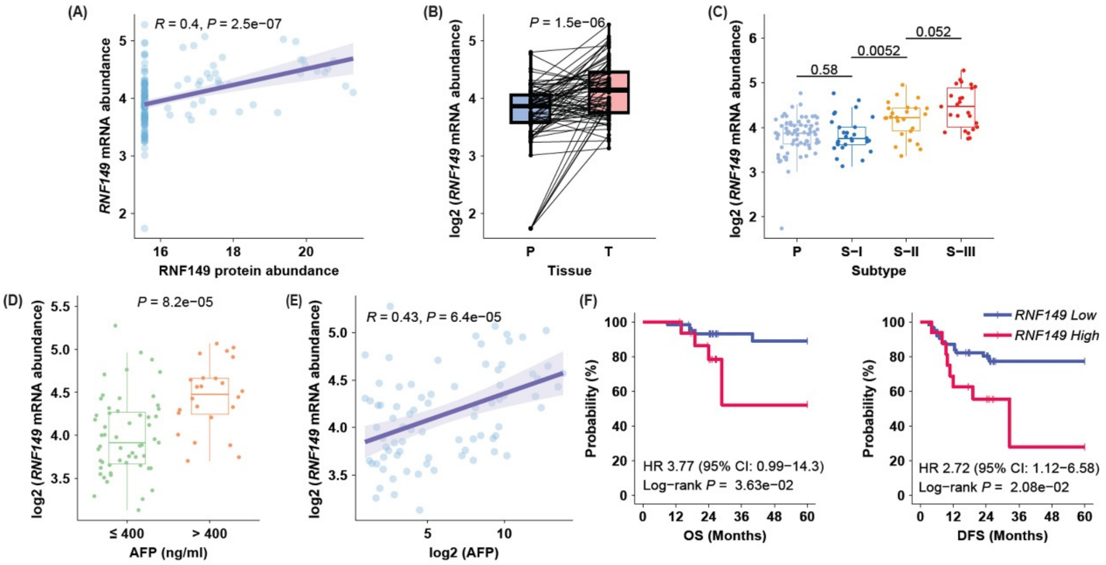

Supplementary Figure S2. The mRNA expression of RNF149 is positively correlated with the protein abundance, and high mRNA level of RNF149 expression is correlated with poor prognosis of HCC. (A) Correlation analysis between the mRNA and protein expression level of RNF149. (B) Expression of RNF149 mRNA in HCC tissues and paracancerous tissues. (C) RNF149 mRNA expression among three subtypes of HCC tissues. (D) RNF149 mRNA expression in AFP-low (≤400 ng/mL) and AFP-high (>400 ng/mL) groups. (E) Correlation analysis between the serum AFP levels and RNF149 mRNA expression. (F) Kaplan-Meier survival analysis for OS and DFS of HCC patients with different RNF149 mRNA expression under the optimal cut-off value.

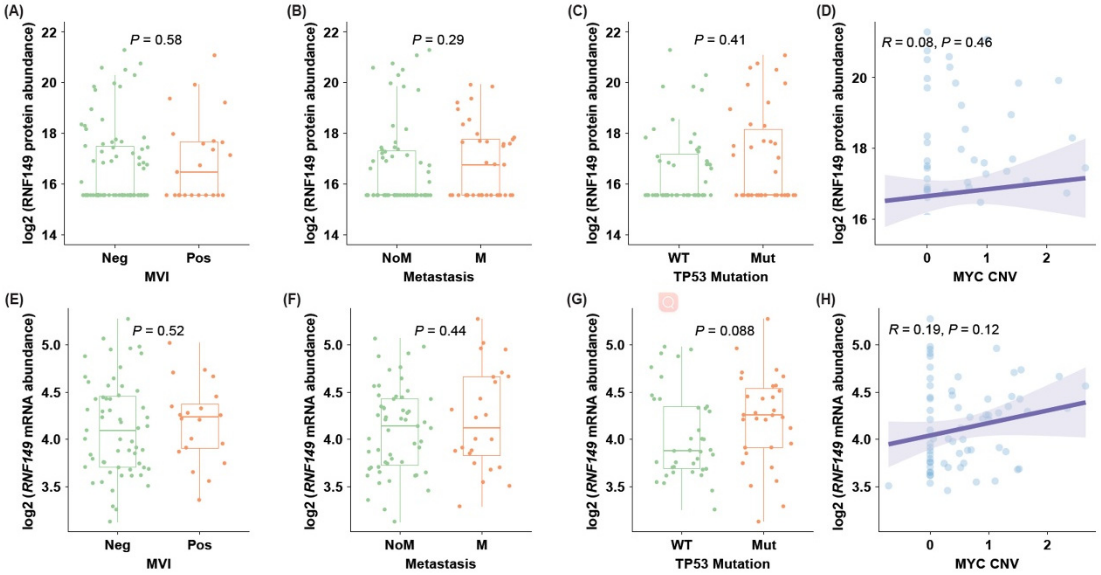

Supplementary Figure S3. The correlation between the RNF149 protein and mRNA expression and MVI, metastasis, TP53 mutation, MYC amplification. (A) RNF149 protein expression in MVI positive and negative groups. (B) Difference of RNF149 protein expression between metastasis and non-metastasis groups. (C) Expression of RNF149 protein in TP53 mutant and non-mutant group. (D) Correlation analysis between the RNF149 protein expression and MYC copy number variation. (E) RNF149 mRNA expression in MVI positive and negative groups. (F) Difference of RNF149 mRNA expression between metastasis and non-metastasis groups. (G) Expression of RNF149 mRNA in TP53 mutant and non-mutant group. (H) Correlation analysis between the RNF149 mRNA expression and MYC copy number variation.

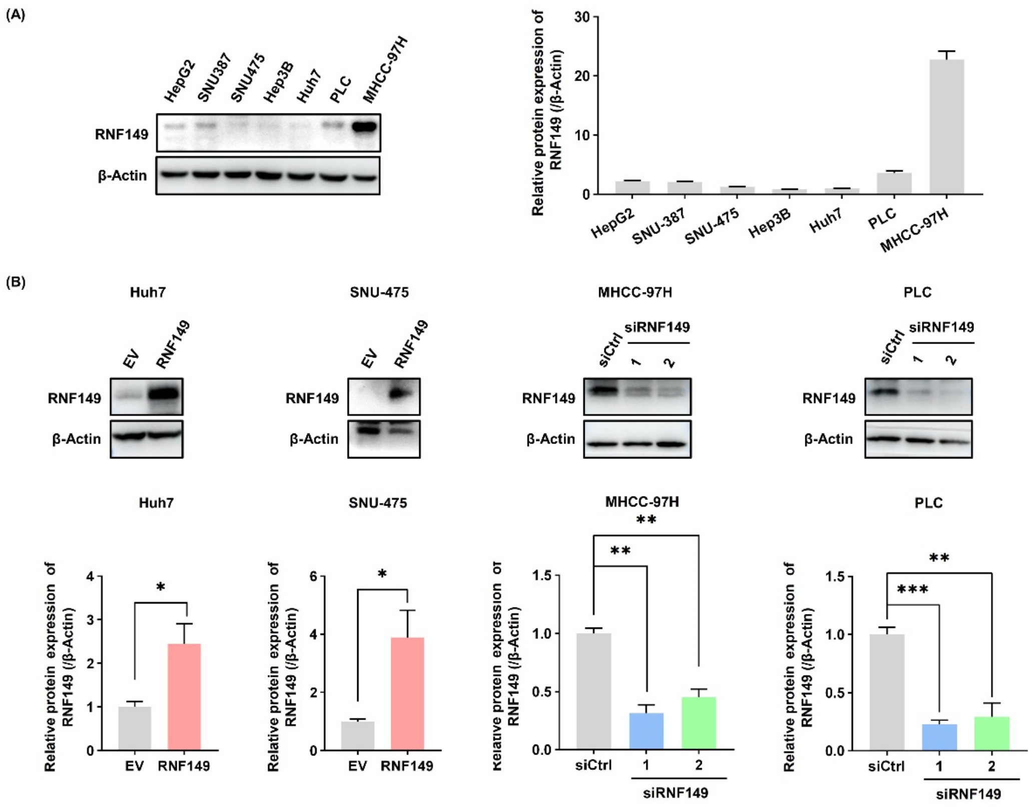

Supplementary Figure S4. The basal expression of RNF149 in multiple HCC cell lines and the efficiency of RNF149 overexpression or knockdown in cells. (A) The basal protein expression of RNF149 in different HCC cell lines was detected by WB. (B) Huh7, SNU-475, MHCC-97H and PLC cells were transfected with indicated plasmids or siRNAs, and RNF149 levels were detected by WB. *$P < 0.05$, **$P < 0.01$, ***$P < 0.001$.

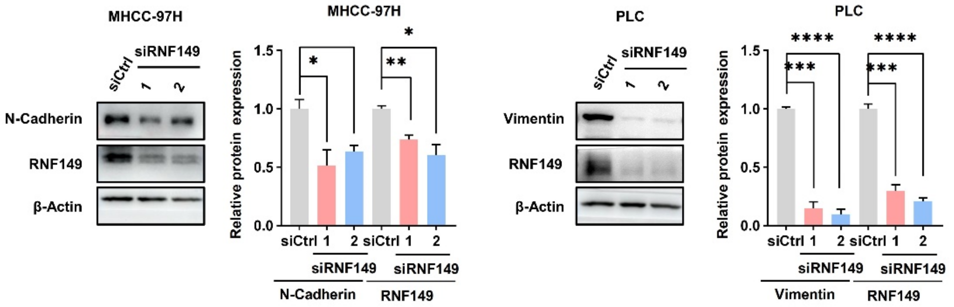

Supplementary Figure S5. Knockdown of RNF149 resulted in decreased EMT in HCC cells. MHCC-97H and PLC/PRF/5 cells were transfected with indicated siRNAs and EMT marker was determined using WB. *$P < 0.05$, **$P < 0.01$, ***$P < 0.001$,****$P < 0.001$.

Whole Western Blot Figures

Figure 5B

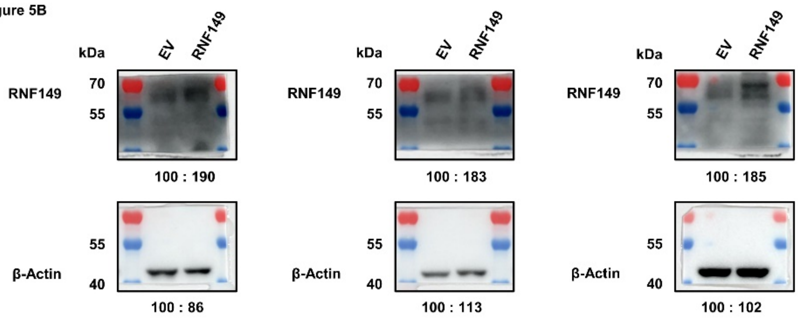

Figure 6A

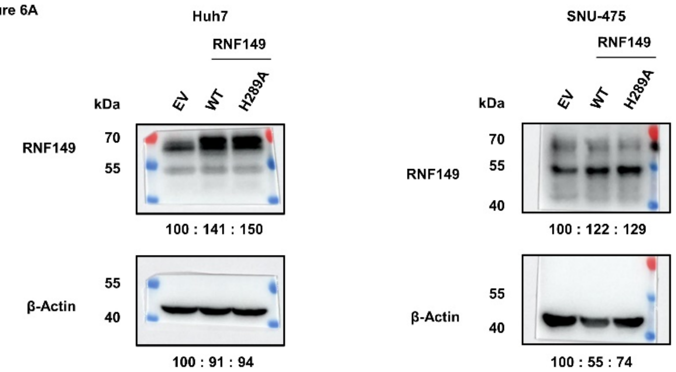

Figure 7H

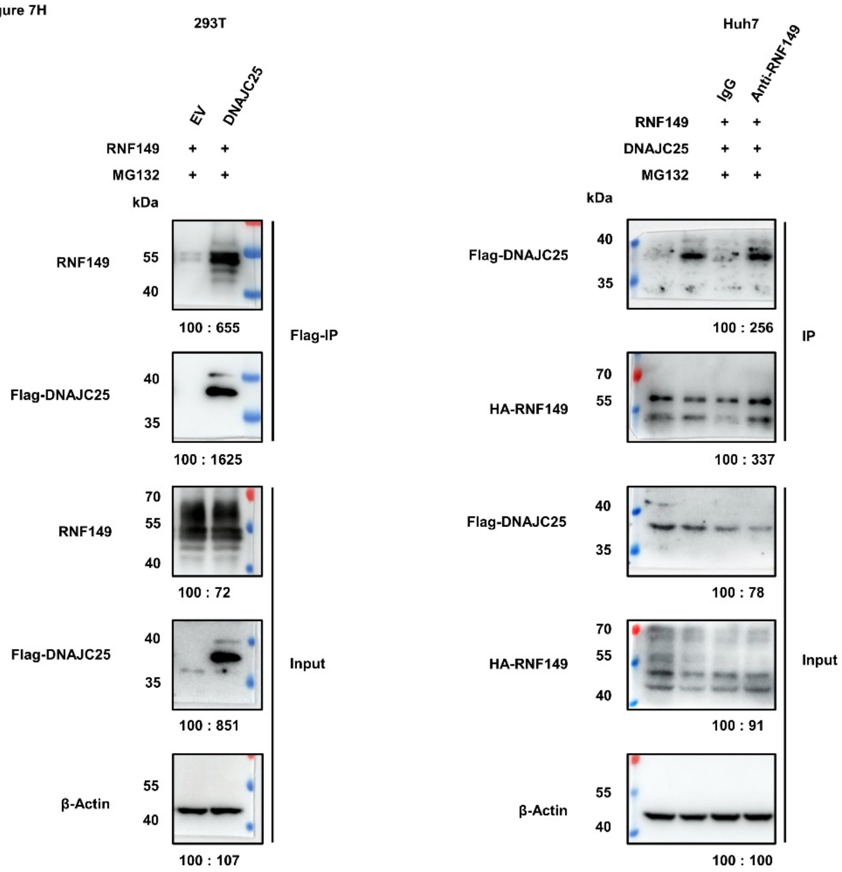

Figure 7I

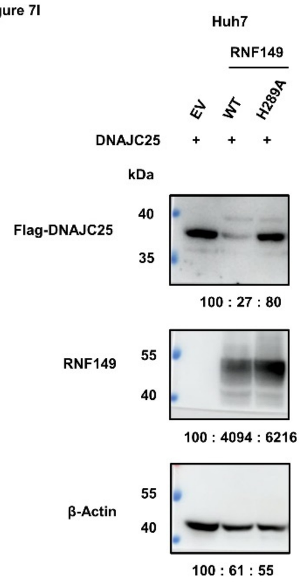

Figure 7J

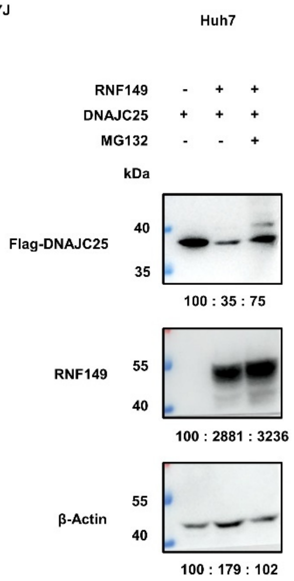

Figure S4A

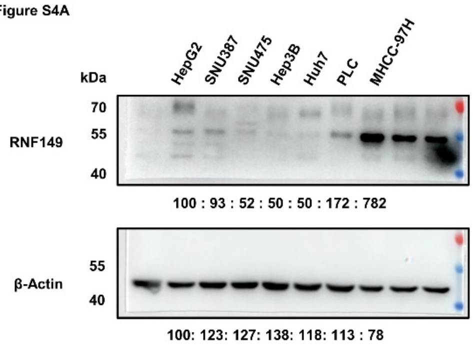

Figure S4B

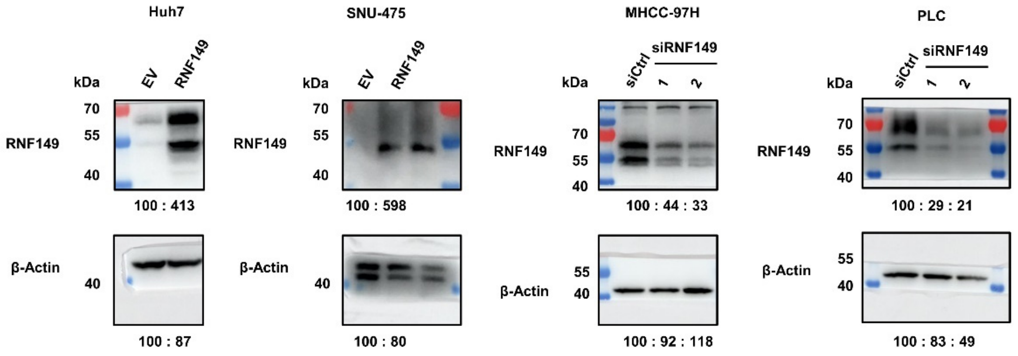

Figure S5

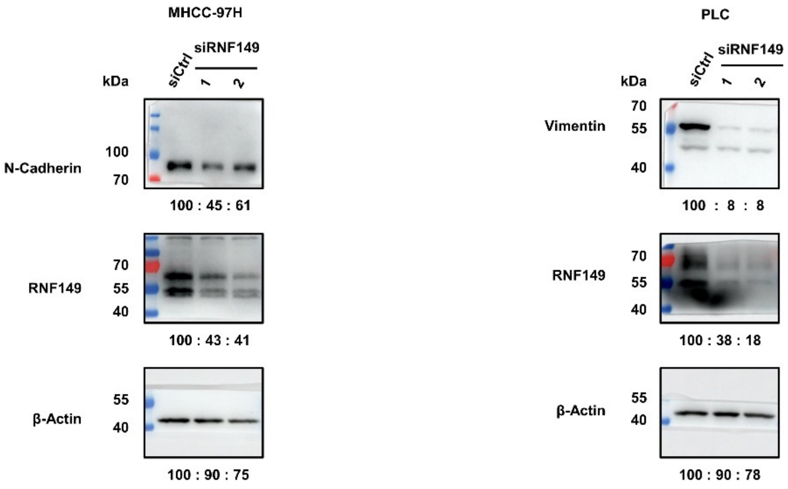

Supplement: Supplementary file 1 [file cancers-15-05203-s001.zip › cancers-2670600-supplementary.pdf]
